# Binding Affinity Determination in Drug Design: Insights from Lock and Key, Induced Fit, Conformational Selection, and Inhibitor Trapping Models

**DOI:** 10.3390/ijms25137124

**Published:** 2024-06-28

**Authors:** Danislav S. Spassov

**Affiliations:** Drug Design and Bioinformatics Lab, Department of Chemistry, Faculty of Pharmacy, Medical University of Sofia, 1000 Sofia, Bulgaria; dspassov@pharmfac.mu-sofia.bg

**Keywords:** binding affinity, inhibitor potency, lock and key, induced fit, conformational selection, inhibitor trapping, Abl, imatinib, RAS, kinase, magic methyl, drug design

## Abstract

Binding affinity is a fundamental parameter in drug design, describing the strength of the interaction between a molecule and its target protein. Accurately predicting binding affinity is crucial for the rapid development of novel therapeutics, the prioritization of promising candidates, and the optimization of their properties through rational design strategies. Binding affinity is determined by the mechanism of recognition between proteins and ligands. Various models, including the lock and key, induced fit, and conformational selection, have been proposed to explain this recognition process. However, current computational strategies to predict binding affinity, which are based on these models, have yet to produce satisfactory results. This article explores the connection between binding affinity and these protein-ligand interaction models, highlighting that they offer an incomplete picture of the mechanism governing binding affinity. Specifically, current models primarily center on the binding of the ligand and do not address its dissociation. In this context, the concept of ligand trapping is introduced, which models the mechanisms of dissociation. When combined with the current models, this concept can provide a unified theoretical framework that may allow for the accurate determination of the ligands’ binding affinity.

## 1. Introduction

One of the primary aims of computational research on drug-target interactions is the estimation of binding affinity to differentiate between strong and weak binding or nonbinding molecules targeting a specific protein [1]. One of the most frequently used computational approaches to studying protein-ligand interactions is docking [2,3,4,5,6,7]. Docking has two main goals—(i) predicting the position of the ligand in the binding site and (ii) predicting the binding affinity, which is expressed as a scoring function—a mathematical model used to evaluate and quantify the strength of interactions between a protein and a small molecule ligand [3,5]. The scoring function considers various factors such as van der Waals interactions, electrostatic interactions, hydrogen bonding, desolvation effects, and entropic contribution, and its goal is to rank different ligands based on their binding affinity, thereby aiding in the identification of potential drug candidates [5,8]. In general, docking produces excellent results in identifying the correct pose of the ligand, demonstrating a remarkable agreement with the crystal structures of the corresponding protein-ligand complexes; however, the scoring functions have been found to be uncorrelated with the experimentally determined binding affinity and activity [9,10,11,12,13,14]. In the search for better scoring functions, different methodologies have been developed [8]. The empirical scoring functions are based on estimating the strength of the interactions, determined through datasets of experimental results, and have gained broader application in the most popular docking programs, such as Autodock, Glide, GOLD, and MOE [15,16,17,18,19]. Additionally, force field-based scoring functions, exemplified by Molecular Mechanics/Generalized Born Surface Area (MM/GBSA) and Molecular Mechanics/Poisson-Boltzmann Surface Area (MM/PBSA), employ sophisticated calculations to model the detailed atomistic interactions between the ligand and protein during molecular dynamics simulations [20]. Knowledge-based scoring functions, including linear regression models and machine learning algorithms, leverage data from known protein-ligand complexes to predict binding affinities based on structural features [15,21].

Despite advancements in computational tools for drug design, accurately predicting binding affinity remains an elusive goal, with predicted values often diverging by orders of magnitudes from experimental results [9,10,11,12,13,14]. This can be attributed to two plausible reasons. One is that the scoring functions may inaccurately estimate factors such as bond strength, solvation effects, entropy, or polarization, thereby providing results that are not consistent with the experimental measurement [10,22,23,24]. This belief has fueled the development of the different scoring functions, but it has not produced satisfactory results yet. The second explanation suggests that computational methods may not accurately or comprehensively model the biological and chemical mechanisms that determine binding affinity in the first place. Indeed, current models such as the lock and key, induced fit, and conformational selection (outlined in Section 3), upon which computational tools are based, primarily focus on the binding step during complex formation but do not model the dissociation rate of the ligand. This shortcoming could result in the inability of computational approaches to estimate binding affinity. For instance, the ligand trapping mechanism, recently reported in *N*-myristoyltransferases and kinases, results in a dramatic increase in binding affinity [14,25]. However, this mechanism is not considered in any existing computational tools for affinity prediction. Therefore, developing computational tools capable of estimating the degree of ligand entrapment and the dissociation rate, respectively, could unlock the potential for accurate estimation of binding affinity in protein-ligand complexes.

The following sections outline the basic definitions of binding affinity and rate constants and how they are linked with the current models of protein-ligand recognition. Subsequently, the inhibitor trapping model will be described, providing examples and evidence for its existence and discussing its impact on binding affinity. In the final section, the various models will be consolidated into a unified framework, and their potential applications in drug design will be discussed.

## 2. Binding Affinity, Binding and Dissociation Rate Constants 

In everyday usage, “affinity” typically refers to a natural liking, attraction, or inclination towards something or someone. When describing protein-ligand interactions, binding affinity is frequently equated to the strength of the interaction between the protein and ligand [1]. However, the term “binding affinity” originates from kinetics and is indicated by the affinity constant *K_a_*, which arises at the equilibrium of the binding and dissociation rates of the interaction between a protein and a ligand [26]. In this context, binding affinity measures the stability of the protein-ligand complexes rather than the mere attractiveness or strength of the interaction. Given the various interpretations of “affinity”, the mathematical definition and derivation of the binding affinity constant in kinetics are provided below:

The formation of a ligand-protein complex is a two-state process that depends on the rate of association (on rate) and dissociation of the complex (off rate) [26]. For the interaction, where *L* represents the ligand, *P* the protein, and *LP* the ligand-protein complex:

$$L+P\overset{\mathrm{on}}{\underset{\mathrm{off}}{⇌}}LP$$

The speed of the on and off reactions (*V_on_* and *V_off_*) is given by Equations (1) and (2).

(1) $$V_{on}=k_{on}\;\left[L\right]\left[P\right]$$

(2) $$V_{off}=k_{off}[LP]$$

where [*L*], [*P*], and [*LP*] represent the molar concentrations of the protein, ligand, and protein-ligand complex, respectively. The *k_on_* is the association (binding) rate constant, expressed in M^−1^s^−1^, and *k_off_* is the dissociation rate constant, expressed in s^−1^. 

When equilibrium is reached, *V_o_*_n_ = *V_off_*

(3) $$k_{on}\;\left[L\right]\left[P\right]=k_{off}\left[LP\right]$$

or

(4) $$\frac{k_{on}}{k_{off}}=\frac{\left[LP\right]}{\left[L\right][P]}$$

The affinity constant *K_a_* is defined as the ratio between the on and off-rate constants.

(5) $$K_{a}=\frac{k_{on}}{k_{off}}=\frac{\left[LP\right]}{\left[L\right][P]}$$

Therefore, the determination of binding affinity requires an assessment of both the rate of association and the rate of dissociation. This distinction is crucial because binding affinity cannot be estimated solely by the rate of the binding step of the interaction. Hence, when estimating binding affinity, it will be necessary to provide an independent assessment for both ligand binding and dissociation. 

The dissociation constant *K_d_* is reciprocal to the affinity constant *K_a_*, according to Equations (6) and (7).

(6) $$K_{d}=\frac{1}{K_{a}}$$

(7) $$K_{d}=\;\frac{k_{off}}{k_{on}}=\frac{\left[L\right][P]}{\left[LP\right]}$$

*K_d_* is the concentration of the ligand at which 50% of the protein is occupied. This can be derived from Equation (7)—when ([*P*] = [*LP*]), then *K_d_* = [*L*]. *K_d_* has the dimensions of concentration and is expressed in molarity (M) units, making it preferable to the affinity constant *K_a_* in the scientific literature. 

When considering enzyme-inhibitor complexes, instead of the dissociation constant *K_d_*, frequently reported in the literature, is the inhibition constant *K_i_*. *K_i_* represents the dissociation constant for the binding of an inhibitor to a protein. *K_i_* is typically used whenever the equilibrium constant is measured through inhibition kinetics, whereas *K_d_* is preferred when the constant is determined more directly, for example, by isothermal titration calorimetry or surface plasmon resonance [27].

Ultimately, the binding affinity is dictated by the mechanisms governing the interaction between the protein and the ligand. These will be explored in the upcoming section. 

## 3. Models of Protein-Ligand Recognition

**Figure 1:**
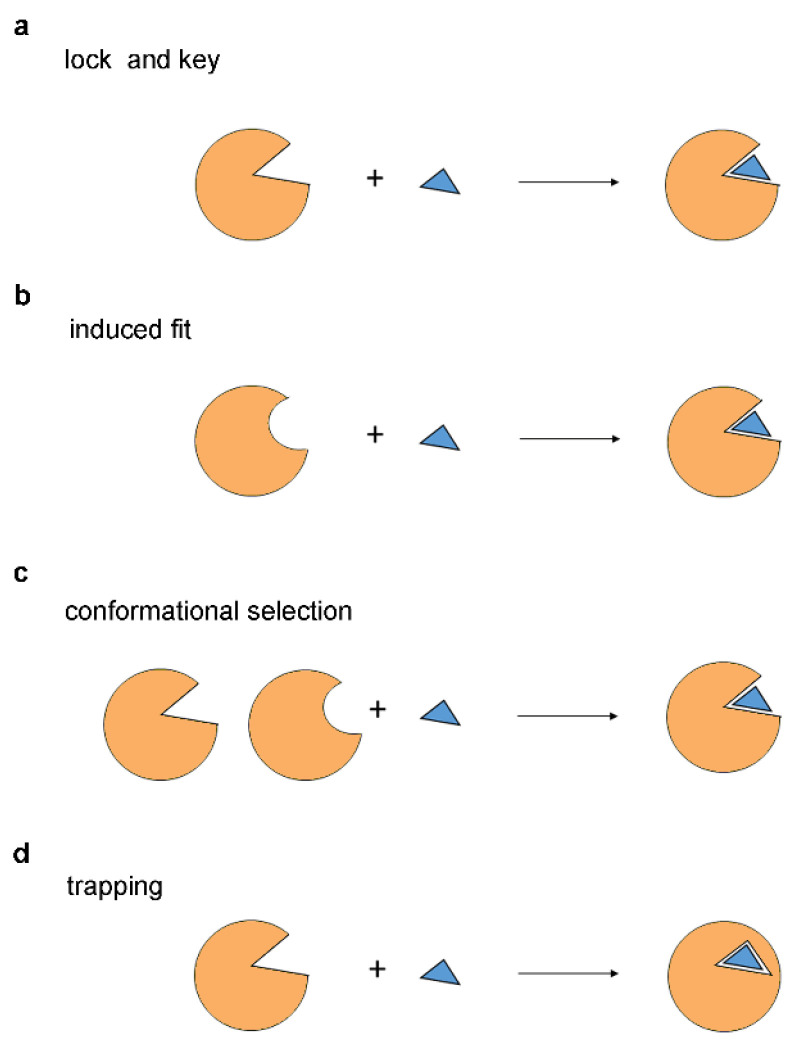
Models of protein-ligand interaction. (**a**) In the lock and key model, the ligand fits precisely into the active site of the protein and has a complementary shape. (**b**) In the induced fit model, the protein undergoes conformational changes to accommodate the binding of the ligand. (**c**) In the conformational selection model, the protein can exist in different conformations, and the ligand selectively binds to a specific conformation. (**d**) In the trapping model, the ligand is captured inside the confines of the protein structure. The protein is shown in orange, and the ligand in blue.

The fundamental principles of protein-ligand interactions have emerged from early investigations into enzyme-substrate recognition. The first model of enzyme-substrate binding, the lock and key model lock (Figure 1a), which was proposed by Emil Fischer in 1894, suggests that the substrate has a shape complementary to that of the enzyme catalytic site, akin to a key fitting into a lock [28]. It implies that only substrates with precisely matching shapes can bind to the enzyme. The lock and key model, initially devised to explain how enzymes selectively recognize and bind to specific substrates or their stereoisomers, has been extrapolated to elucidate interactions between inhibitors and enzymes, as well as between proteins and ligands in general. Over time, the lock and key model has become one of the most prominent and enduring paradigms, providing valuable insights into the mechanisms underlying molecular recognition [26].

With the advent of crystallographic analysis, it has become evident that the lock and key model provides an overly simplistic view of protein-ligand interactions. In particular, in the lock and key model, the protein and the ligand are portrayed as rigid molecules. In reality, the ligand can adopt different conformations depending on the number and positions of its rotatable bonds, and the proteins are highly flexible and capable of shifting their shape and topology [29]. The induced fit model (Figure 1b), proposed by Daniel Koshland in 1958, suggests that the ligand structure may not be perfectly complementary to the binding site, but as they interact, the protein adjusts its shape to achieve a better fit, akin to putting a hand into a glove [30,31]. Today, it is widely accepted that proteins can undergo conformational changes upon binding to a ligand, which is in agreement with the induced fit theory. These conformational changes have been described as hinge domain and shear motions, and their magnitude could be substantial or subtle depending on the protein-ligand complex [32]. Initially intended to explain the recognition between enzymes and substrates, the induced fit models have gained acceptance for explaining the recognition between proteins and ligands in general.

For over half a century, the induced fit hypothesis has remained the textbook explanation for molecular recognition events; however, in 2009, an alternative model was proposed by David Boehr, Ruth Nussinov, and Peter Wrigh that has become known as the conformational section theory [33]. According to the conformational selection model, the proteins can exist in multiple conformational states, and the ligand selects or stabilizes the most favored conformation (Figure 1c) [33]. The conformational selection offers an alternative to the induced fit theory, suggesting that enzymes or proteins, in general, possess an intrinsic ability to transition between different conformations while the ligands selectively interact with a specific pre-existing conformation [34]. In this model, the role of the ligand is not to induce the conformational change but to stabilize the particular conformation it selects. Conformational selection theory has gained prominence in the kinase field, for example, where the crystallographic structures have revealed that the different inhibitors interact with diverse active and inactive conformations and the realization that the kinase domain, can adopt these various conformations, even in the absence of the ligand [35]. In general, conformational selection has been observed for protein-ligand, protein-protein, protein-DNA, protein-RNA, and RNA-ligand interactions [33].

While the lock and key, induced fit, and conformational selection models have gained widespread acceptance, we recently observed that these models exhibit limitations in explaining the mechanism of action of certain *N*-myristoyltransferase and kinase inhibitors [14,25]. Specifically, these models are focused on ligand binding without addressing ligand dissociation, making them incomplete for predicting inhibitor binding affinity or activity. To address this, we recently proposed the inhibitor trapping model [14,25]. In the inhibitor trapping model, a ligand binds to an open protein conformation and is subsequently captured and trapped inside the binding site in a closed conformation of the protein (Figure 1d). The trapping mechanism prevents the dissociation of the ligand, thus reducing the dissociation rate constant *k_off_*. As described in this article, this mechanism can increase the binding affinity by thousands or even millions of times (for example, in the streptavidin-biotin complex). 

## 4. The Inhibitor Trapping Mechanism

**Figure 2:**
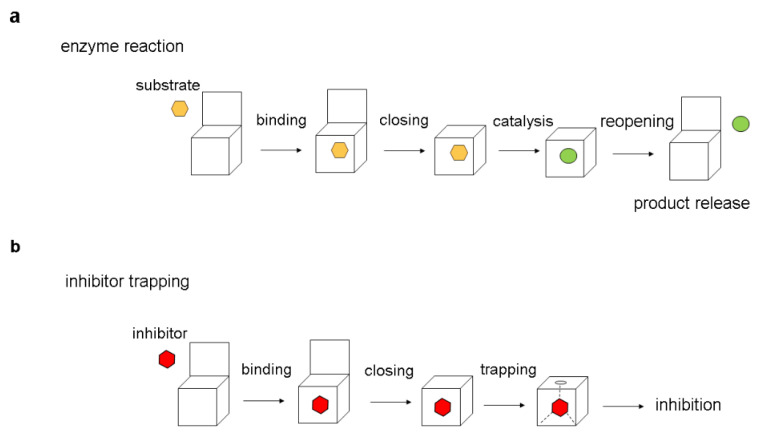
A schematic representation of inhibitor trapping. (**a**) Events during normal enzyme reactions. (**b**) Events leading to inhibitor entrapment. The trapping occurs as a result of blockage of the transition between closed and open conformation, preventing the dissociation of the small molecule. The open and closed enzyme conformations are depicted as open or closed boxes. The substrate is shown in orange, the product in green, and the inhibitor in red.

Many enzymes are known to undergo conformational changes during catalytic reactions. Typically, substrates bind to enzymes in their open conformation, where the catalytic center is readily accessible. Subsequently, the catalytic reaction occurs in a closed enzyme form, and the enzyme must reopen to release reaction products (Figure 2a). The inhibitor trapping mirrors certain aspects of this process. Similarly, inhibitors initially bind to an open enzyme conformation and subsequently become confined to the active site upon the establishment of the closed enzyme conformation. One key difference is that once the inhibitor enters the enzyme’s active site, it participates in interactions that block the reopening of the enzyme, leading to the entrapment of the small molecule inside the enzyme structure (Figure 2b).

While the role of the open conformation is clearly to allow the binding of the substrate and the release of the product of the reaction, why does the enzyme need to adopt a closed conformation in the first place? The closed enzyme conformation is the catalytically active form, and its formation could be required for the proper alignment of the amino acid residues within the enzyme active site, which is necessary for efficient catalysis [30]. The closing of the enzyme may also be needed to exclude water molecules from the catalytic center. For instance, the hydroxyl groups of the water molecules are as reactive as the hydroxyl groups present in some substrates, such as peptides or sugars, and therefore could interfere with certain enzyme reactions [30]. In kinases, the inability to exclude water from the catalytic center may lead to the hydrolysis of ATP (ATPase activity) instead of phosphorylating the substrate of the reaction [30]. The capture of the substrate in its enzyme-closed form may also be required to extend its residence time at the active site, facilitating catalysis. In support of this notion, fast substrate dissociation rates are typically correlated with poor catalytic rates [36]. Hence, in general, the enzymes may have evolved to transit from open to closed conformations and vice versa, allowing the proper substrate binding, catalysis, and product release.

There are two distinct possibilities for how the transition from open to closed conformation could occur. In the first, the binding of the substrate may induce the conformational change to a closed enzyme conformation (induced fit), and in the second, this is due to the intrinsic capacity of the enzyme to do so (conformational selection). Therefore, the ligand entrapment model incorporates the induced fit and conformational selection theories to describe the binding of the ligand to the open enzyme conformation.

**Figure 3:**
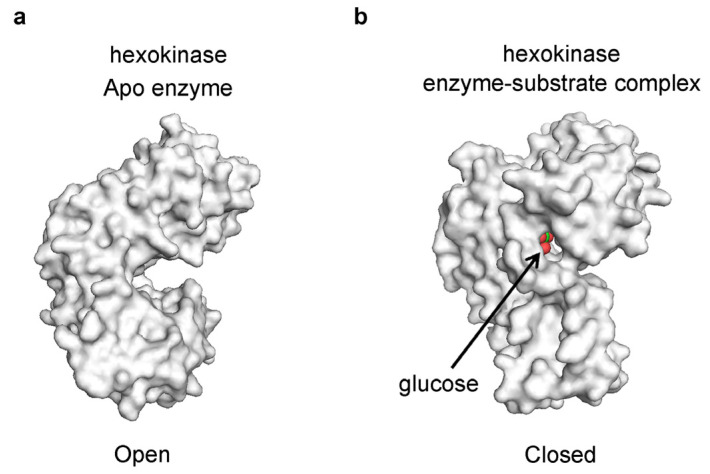
Substrate trapping in hexokinase (**a**) The crystal structure of hexokinase in the Apo form (PDB 2E2N) shows the enzyme in an open conformation. (**b**) The crystal structure of hexokinase in complex with glucose (PDB 2E2O) shows the enzyme in closed conformation with the glucose molecule captured inside the enzyme active site. The carbon atoms of glucose are colored in green and the oxygen atoms in red. The images depict surface representations of the crystal structures and were produced using PyMOL v1.6.0.0 [40].

The existence of open and closed conformations has been observed in the crystal structures of many enzymes; however, these have been mainly attributed to induced fit binding of the substrate. For example, in the absence of a substrate, the enzyme hexokinase adopts an open conformation, but upon binding of the substrate, it closes, capturing the substrate in the catalytic center (Figure 3) [30,37]. Hexokinase catalyzes the transfer of a phosphate from ATP to the hexoses—the six-carbon monosaccharides, including glucose and fructose, among others [38]. Among its many functions, most notably, hexokinase is responsible for the phosphorylation of glucose in the first step of glycolysis [39]. Crystallographic structures have revealed that, in the absence of a substrate, the hexokinase adopts an open conformation where the catalytic site is fully accessible (Figure 3a). However, in the presence of glucose, the enzyme transitions to a closed conformation, capturing the glucose molecule within the protein structure (Figure 3b) [37].

A similar closed structure has been observed in the complex between hexokinase and xylose (PDB 2E2Q) [37]. Xylose is a pentose—a five-carbon monosaccharide that potently inhibits the kinase activity of hexokinase but increases the capacity of the enzyme to hydrolyze ATP (or its ATPase activity) [30,41]. The existence of the closed enzyme conformation in these crystal structures has been reported in the literature to be a result of induced fit binding, i.e., that the substrate binding induces a conformational change in hexokinase [37,42,43,44]. However, although not apparent at the time, these experiments also provide evidence of ligand entrapment, as only by forming a stable complex with hexokinase can xylose effectively block new glucose molecules from accessing the catalytic center to inhibit the enzyme activity. This is an excellent example of how a compound, highly structurally related to the substrate, can interfere with the conformational transitions in the enzyme, leading to inhibition through entrapment. 

**Figure 4:**
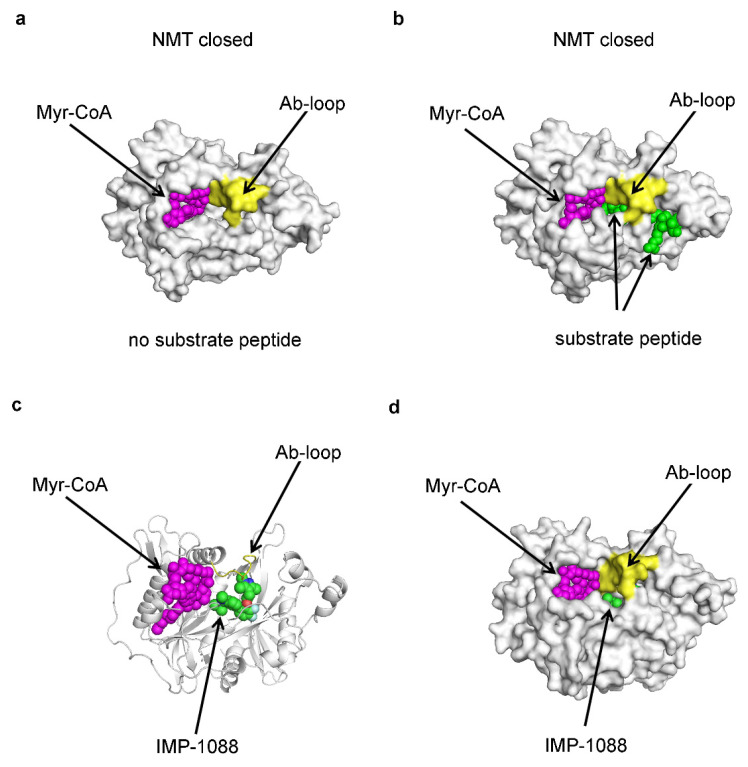
Inhibitor trapping in *N*-myristoyltransferase. (**a**) The crystal structure of the binary complex between human NMT1 protein and its cofactor, Myr-CoA (PDB 3IU1), shows the enzyme in a closed conformation. (**b**) The crystal structure of the ternary complex of human NMT1 protein, Myr-CoA, and substrate peptide (PDB 6QRM) shows the enzyme in closed conformation with the substrate peptide (green) located inside the enzyme active site. (**c**,**d**) The crystal structure of human NMT1 in complex with the inhibitor IMP-1088 is shown (PDB 5MU6). (**c**) A cartoon representation depicting IMP-1088 (in green) inside the binding side. (**d**) A surface representation shows that the inhibitor is almost completely buried inside the enzyme catalytic center. The carbon atoms of IMP-1088 are shown in green, and nitrogen and oxygen atoms are shown in blue and red, respectively. The Ab-loop is shown in yellow, and Myr-CoA in magenta. Images were produced in PyMOL v1.6.0.0 [40].

In N-myristoyltransferases (NMT), the unliganded enzyme adopts a closed conformation similar to the one observed in the liganded form (Figure 4a–d), implying that the closed enzyme conformation is formed by an intrinsic conformational transition in the enzyme structure, rather than induced fit [14,45]. NMT catalyzes the transfer of myristic acid (a 14-carbon fatty acid), using Myristoyl-Coenzyme A (Myr-CoA) cofactor, to the N-terminus of specific proteins, facilitating the association of the modified proteins with the cellular membranes [14,45,46,47]. NMT recognizes the N-terminal region of the proteins that myristoylate, and in vitro, the reaction can be carried out using the corresponding peptide (substrate peptide) [45]. The crystal structures of NMT in the absence of ligands, in complex with a substrate peptide or NMT inhibitors, have revealed that the enzyme adopts a similar closed conformation (Figure 4a–d) [45]. In all these complexes, the Ab-loop in the NMT catalytic domain adopts a closed conformation that sterically blocks the enzyme catalytic site (Figure 4a–d) [45]. Since the substrate peptide or the inhibitors are incapable of entering the enzyme in this closed enzyme conformation, the initial interaction should involve an alternative open Ab-loop conformation followed by the closing of the loop after the ligand entry [45]. 

In the case of the substrate peptide, the myristoylation reaction proceeds, and the products of the reaction are released after the reopening of the Ab-loop [45]. In contrast, the NMT inhibitors interact with residues at the base of the Ab-loop and prevent its reopening, effectively trapping themselves inside the catalytic center [14]. The entrapment of the NMT inhibitors leads to a very low dissociation constant (for example, *K_d_* = 0.210 nM for IMP-1088) due to a slow rate of dissociation (*k_off_* = 1.9 × 10^−4^ s^−1^) [47].

Molecular dynamics simulations have confirmed that, in NMT complexes with highly potent inhibitors (such as IMP-10088 and DDD85646), the Ab-loop remains locked into the closed conformation and cannot reopen [14]. In contrast, the opening of the Ab-loop was observed in the absence of inhibitors or in NMT complexes, with inhibitors exhibiting low activity [14]. Additionally, in NMT complexes with compounds of low potency, conformational changes occurred in other regions of the protein [14]. A correlation was observed between the degree of conformational dynamics and the potency of the compound, indicating that stabilizing the closed enzyme conformation is essential for effective inhibition [14]. 

**Figure 5:**
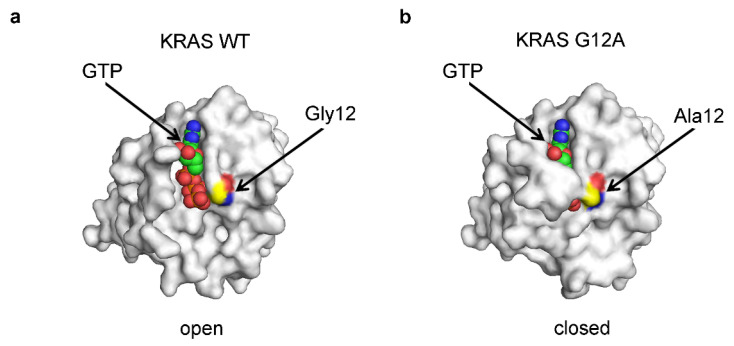
GTP trapping in KRAS G12A mutant. (**a**) The crystal structure of the human WT KRAS protein complex with GTP (PDB 5VQ2). (**b**) The crystal structure of the human KRAS G12A mutant protein complex and GTP (PDB 5VP1). The carbon atoms of GTP are shown in green, and the Gly12 or Ala12 are shown in yellow. Nitrogen and oxygen atoms are shown in blue and red, respectively. Images were produced in PyMOL v1.6.0.0 [40].

Inhibiting the catalytic reaction may impede product formation and subsequent reopening of the enzyme necessary for product release, resulting in stable entrapment of the substrate within the catalytic center. Such substrate trapping may occur as a result of mutations in the RAS proteins. The three RAS genes in the human genome—KRAS, HRAS, and NRAS—are among the most frequently mutated genes in cancer [48,49,50]. In KRAS, mutations are most commonly observed in codon 12 (for instance, G12D or G12A), codon 13, or codon 61 [49]. Central to RAS function is its ability to bind and hydrolyze guanosine triphosphate (GTP) [51]. RAS acts as a molecular switch, cycling between an inactive GDP-bound state and an active GTP-bound state [51]. The RAS mutations reduce its capacity to hydrolyze GTP, thus leaving the molecule in the ‘on’ GTP-bound state, leading to uncontrollable cellular growth and cancer [51]. In the crystal structure of the KRAS WT complex with GTP, the protein adopts an open conformation, while the KRAS G12A mutant protein adopts a closed conformation, wherein the GTP seems to be captured and trapped inside the structure of the enzyme (Figure 5) [52]. The formation of this closed form stabilizes the constitutively active GTP-bound form and may represent a challenge in the attempts to target RAS with small-molecule inhibitors [53].

**Figure 6:**
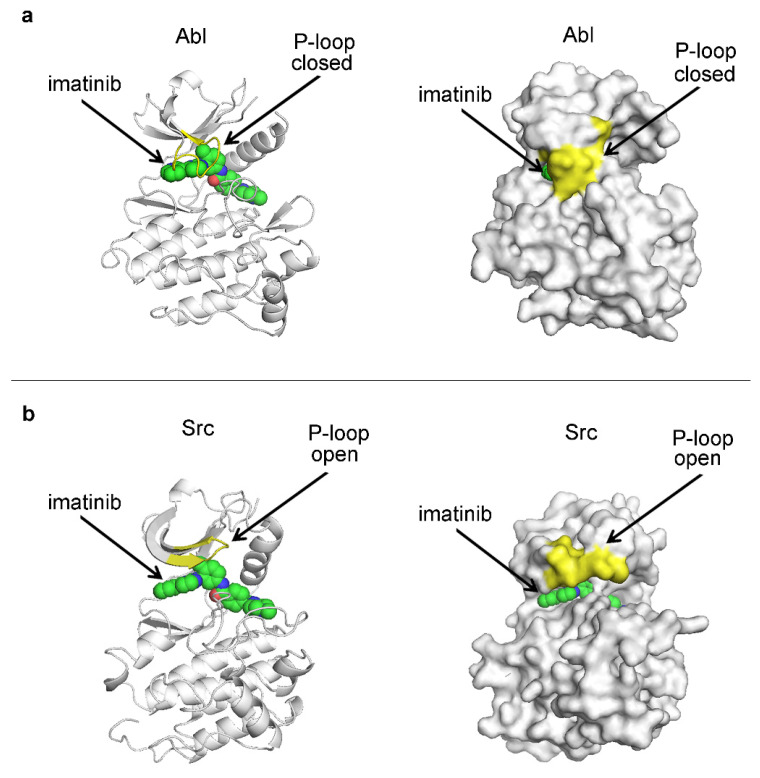
Inhibitor trapping in the Abl-imatinib complex. Images on the left depict cartoon representations of the proteins, allowing visualization of the inhibitor (imatinib) inside the binding side. Surface representations shown in the images on the right are informative about the enclosure (trapping) of the inhibitor. (**a**) Crystal structure of Abl in complex with imatinib (PDB 1IEP). (**b**) Crystal structure of Src in complex with imatinib (PDB 2OIQ). The carbon atoms of imatinib are shown in green, and nitrogen and oxygen atoms are shown in blue and red, respectively. The P-loop is shown in yellow. In Abl, the P-loop adopts a closed conformation, effectively trapping the small molecule inside the catalytic center. Images were produced in PyMOL v1.6.0.0 [40].

Inhibitor trapping is also observed in protein kinases [25]. The catalytic domain of protein kinases contains two lobes connected with a flexible hinge region, which allows the enzyme to open and close [54]. The catalytic center is positioned in the cleft, between the two lobes, and accommodates the adenosine triphosphate (ATP) and the substrate peptide, which is phosphorylated during the kinase reaction [54]. The kinase inhibitors typically bind into the ATP binding pocket [54,55,56]. On top of this pocket is the phosphate-binding loop, also known as the P-loop (Figure 6) [25,57]. In the complex between Abl kinase and imatinib, a drug that has revolutionized the treatment of chronic myeloid leukemia (CML), the P-loop adopts a closed conformation and encloses the inhibitor inside the catalytic center (Figure 6a) [58]. In contrast, in the Src imatinib complex, the P-loop adopts an open conformation, and the inhibitor remains exposed to the solvent (Figure 6b) [59]. 

Imatinib has a 3000-fold higher affinity for the Abl protein over Src (*K_i_* = 10 nM for Abl, and *K_i_* = 30 µM for Src) [60,61], and evidence indicates that this is due primarily to the trapping of the drug by the P-loop [25]. For example, the dissociation rate of imatinib is 100-fold lower from its Abl complex compared to the Src complex [60,61]. 

In addition, many mutations that confer resistance in patients treated with imatinib are clustered in the P-loop of Abl [62]. Particularly interesting is the Y253F mutation [63], in light of the fact that the hydroxyl group of this tyrosine residue forms a hydrogen bond with the hinge region of the Abl kinase domain, helping to seal imatinib inside the catalytic site, but it does not interact directly with the drug [25]. Hence, the pronounced loss of affinity and potency of imatinib towards the Y253F mutant could be explained by the destabilization of the closed conformation of the P-loop, preventing the efficient trapping of the inhibitor inside the protein structure [25]. 

Molecular dynamics simulations have revealed that the entrapment of imatinib depends on a methyl group present in one of its central aromatic rings [25]. In the absence of this methyl group, the P-loop transits to an open conformation, releasing the trap mechanism [25]. A similar mechanism is observed in p38α kinase, where the removal of the analogous methyl group leads to the opening of the P-loop and is accompanied by a 208-fold loss of affinity [25,64]. Due to the profound contribution of the methyl group to affinity, in the literature, this phenomenon has become known as a “magic” methyl effect [65].

**Figure 7:**
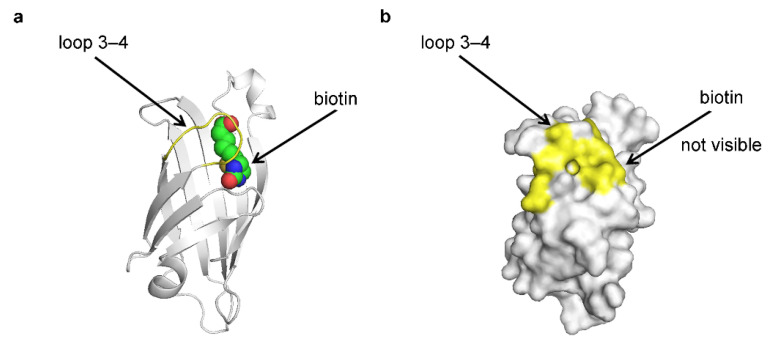
Entrapment of biotin in its complex with streptavidin. The crystal structure of the streptavidin-biotin complex is shown (PDB 1STP). (**a**) A cartoon representation depicting biotin inside the binding side. (**b**) A surface representation shows that the biotin is buried inside the protein structure. The carbon atoms of biotin are shown in green, and nitrogen and oxygen atoms are shown in blue and red, respectively. Loop 3–4, shown in yellow, adopts a closed conformation, effectively trapping the biotin molecule. Images were produced in PyMOL v1.6.0.0 [40].

Entrapment occurs not only in enzymes but also in other protein-ligand complexes. Such is the case with the streptavidin-biotin interaction. The binding of biotin to streptavidin is one of the strongest noncovalent interactions found in nature, with a dissociation constant *K_d_* = 5 × 10^−15^ M [66]. The crystal structure of streptavidin in a complex with biotin reveals a barrel-shaped protein molecule (Figure 7) [67]. The biotin molecule is bound inside the barrel, and it is enclosed within by a flexible loop connecting β-strands 3 and 4, therefore named loop 3-4 (Figure 7) [67]. 

In the crystal structures of the Apo streptavidin, loop 3–4 is either disordered or found in an open conformation [66]. In biotin-bound structures, loop 3–4 is found in the closed conformation and functions as a ‘lid’ over the binding pocket, sterically inhibiting biotin dissociation [66]. Loop 3–4 substantially contributes to the extremely slow biotin dissociation rate, as its removal results in a decrease in binding affinity by millions of folds (*K_d_* = 1 × 10^−7^ M) [66,68]. 

## 5. Inhibitor Trapping in Drug Design

The contribution of inhibitor trapping to binding affinity, dissociation rate, and drug selectivity are discussed in this section. In addition, its role in fragment-based drug design, as well as its effect on protein conformational dynamics and allostery, are also discussed. In some instances, the inhibitor entrapment produces outcomes that are difficult to explain through the conventional models, such as a lock and key, induced fit, and conformational selection. Examples of these include the observations that in some protein-ligand complexes, the direct interactions between the ligand and the protein are not correlated with the binding affinity and activity of the ligands, that some bonds within the protein-ligand complexes are “special” and contribute dramatically by hundreds to thousands of folds to binding affinity, instead of the expected difference of several folds. These experimental observations have puzzled researchers to the point where they’ve described them as “magic” in the literature [69]. Although, these phenomena can be explained by the inhibitor trapping paradigm. The described experimental observations provide compelling evidence for the inhibitor trapping model, as they defy explanation by conventional paradigms of protein-ligand interaction.

### 5.1. Inhibitor Trapping Can Dramatically Enhance the Binding Affinity 

The effect of trapping on binding affinity is substantial. In the case of biotin-streptavidin interaction, the removal of loop 3–4, which participates in trapping the small molecule, reduces the binding affinity by millions of folds [66,68]. In the case of Abl imatinib interaction, the entrapment by the closed P-loop conformation increases the affinity thousands of fold [25]. In NMT, the entrapment increases the potency of the inhibitors by thousands of folds [14]. Hence, without an assessment of ligand entrapment, estimating the binding affinity may prove impossible.

Considering that the trapping creates a steric barrier for dissociation of the small molecule, it primarily affects the dissociation rate constant *k_off_*. The substantially reduced dissociation rate constant leads to increases in the binding affinity, considering the equation *K_a_* = *k_on_*/*k_off_*. As such, low values of the dissociation rate constant *k_off_* can be used as an indication of the presence of entrapment in a given protein-ligand complex. 

Table 1 summarizes experimentally determined values of the dissociation constant *K_d_* and the rate constant *k_on_* and *k_off_* of certain protein-ligand complexes. The entrapment of biotin in its complex with streptavidin is particularly strong, as indicated by the very low value of the rate constant *k_off_* (Table 1, row 1). A high degree of entrapment is evident in the complexes of NMT inhibitors (Table 1, row 2), of imatinib and nilotinib with Abl (Table 1, row 3, 4), and to a lesser extent in the complex between lapatinib and the epidermal growth factor receptor (EGFR) (Table 1, row 7).

The values of the binding and dissociation rate constants *k_on_* and *k_off_* are uncorrelated (Table 1). Some complexes are characterized by a fast rate of binding and slow dissociation (for example, biotin-streptavidin, imatinib—Abl, NMT inhibitors; Table 1, rows 1–4), and others by fast binding and fast dissociation (staurosporine complex; Table 1, row 8). This provides evidence for the existence of an entrapment mechanism that influences the dissociation rate constant independently from the binding rate.

In general, inhibitor trapping could be responsible for tight binding inhibition, a type of inhibition characterized by high affinity due to the ligand’s very low dissociation rate. In addition to NMT, Abl, and biotin-streptavidin complexes, tight binding is also found in many other protein targets, including the JAK kinases, cyclooxygenases COX-1/2, dihydrofolate reductase-DHFR, and enoyl reductase, among others, indicating the occurrence of inhibitor trapping across diverse proteins [72,73,74,75].

However, it may be incorrect to assume that inhibitor trapping occurs only in the cases of tight binding. Rather, this mechanism may operate in any protein-ligand complex, albeit to varying degrees, of which we could be informed by the dissociation rate constant *k_off_*. For example, instances of tight binding may indicate cases where the protein effectively encloses the ligand within its structural confines. In other complexes, however, the closure might be partial, necessitating a lesser degree of conformational movement to enable the opening of the protein and dissociation of the ligand.

A quantitative measure for the level of entrapment can be provided by the residence time—the time that a drug remains bound to its target before dissociating (Table 1) [76]. The residence time is defined as the reciprocal value of the *k_off_* rate constant (residence time = 1/*k_off_*) [76]. Since the residence time is expressed in time units (seconds, for example), its values can be more intuitively understood than those of the rate constant *k_off_*, which is expressed in s^−1^ units. Intriguingly, the inhibitor trapping model suggests a novel interpretation of the residence time. When expressed in seconds, the residence time, in fact, shows how many times the binding affinity increases as a result of the entrapment of the ligand. For example, from the equation *K_a_* = *k_on_*/*k_off_*, it follows that *K_a_* = *k_on_* × residence time. If the residence time = 1 s, then *K_a_* = *k_on_*, i.e., this is the binding affinity in the absence of entrapment. Therefore, an increase in the binding affinity above the value of the binding rate constant *k_on_* is an indication of entrapment. As such, the entrapment of biotin increases the binding affinity over 3.7 million-fold, of imatinib 1706-fold, of staurosporine 33-fold, etc. (see Table 1, column next to the last). Therefore, we propose using residence time, expressed in seconds, as an indicator of entrapment in protein-ligand complexes, with a residence time of 1 s indicating a lack of entrapment (Table 1).

Drugs exhibiting a high degree of entrapment may possess desirable properties for clinical use. For example, the entrapment of imatinib in the Abl kinase could be pivotal to its success in treating chronic myeloid leukemia (CML) [25,77]. Nilotinib, which shows an even higher degree of entrapment (Table 1, compare rows 3 and 4), is effective against the resistant mutations observed in the P-loop of the Abl kinase domain in patients treated with imatinib [70]. The clinical success of drugs that exhibit high entrapment may be attributed to their higher selectivity. For instance, while imatinib can inhibit c-Kit and platelet-derived growth factor receptor (PDGFR) in addition to Abl, it demonstrates a high level of selectivity within the 500-member protein kinase family, and lapatinib inhibits only the kinases of the EGFR subfamily [78,79]. In contrast, staurosporine shows a low degree of entrapment (Table 1, row 8) and displays almost no selectivity against the different protein kinases [80]. As a result, staurosporine exhibits significant off-target toxicity, rendering it unsuitable for clinical use [80].

### 5.2. Trapping Enhances Drug Selectivity 

In the lock and key model, if the binding sites of two or more proteins are conserved or near identical, the development of selective inhibitors that target one of the proteins but not the other(s) could be challenging or even impossible. Such a situation could be desirable when it is needed to target a specific protein among a protein family, for example, a protein kinase, among its 500-member family, or to target a protein from an infectious organism that displays high similarities with its human homologues. However, there are examples where selective targeting of conserved binding sites has been achieved. The anticancer drug imatinib, which has revolutionized the treatment of CML and targeted cancer therapy in general, displays high selectivity among members of the kinase family [78]. For example, although the binding sites of imatinib in the Abl kinase and its closest homologue, c-Src, are almost identical, imatinib displays a 3000-fold higher affinity for Abl than Src, due to the entrapment of the drug through the closure of the P-loop (Figure 6) [25,59,60,61]. Therefore, the inhibitor-trapping mechanism plays a critical role in the selectivity of imatinib and, ultimately, in its clinical success.

In NMTs, trapping has enabled the selective targeting of protozoan NMTs while sparing their human counterparts, despite their near-identical binding sites. For example, an inhibitor of *Leishmania major*’s NMT was developed that displays 215-fold higher affinity compared to its human homologues (*K_i_* = 2 nM and 430 nM, resp.) [81]. The authors have determined that the difference in affinity arises from three amino acid residues near the protein’s C-terminus. These residues do not directly engage in inhibitor binding, but likely influence protein dynamics, contributing to the trapping mechanism [81,82]. More recently, an inhibitor of *Plasmodium vivax* NMT was developed that displays approximately 270-fold increased potency compared to its human orthologue [83]. Hence, the paradigm of inhibitor trapping offers an enticing opportunity for the creation of selective drugs tailored to target conserved binding sites.

### 5.3. The Direct Interactions between the Protein and the Ligand Are Not the Sole Determinant of Binding Affinity

The binding affinity is often assumed to originate from the direct bonding between the ligand and the protein. As a result, it could be theorized that its values could be determined by the cumulative effect of the change in free binding energy caused by the formation of each bond between the protein and the ligand. However, the inhibitor trapping model suggests that determining binding affinity is not as straightforward as this implies. Evidence for this notion can be seen in the fact that binding affinity cannot be deduced from experimentally determined crystal structures, even though in them, every bond can be visualized and its potential contribution to binding affinity accounted for. For example, the binding affinity of imatinib is 3000 times higher towards Abl, compared to its closest homologue, c-Src, yet their crystal structures show a near identical binding mode, where the interactions between the ligand and the protein are conserved [59,60,61,84]. Since the bonds created between the ligand and the protein are basically the same in the two complexes, direct binding clearly does not account for the big difference in affinity. The difference in imatinib affinity is due to the conformation of the P-loop, which is closed in the Abl imatinib complex and open in the Src–imatinib complex [25]. Mutations of Tyr253, located in the Abl P-loop, to phenylalanine result in significant resistance to imatinib, with biochemical assays demonstrating a marked reduction in imatinib affinity [63]. Although, the hydroxyl group of Tyr253, which is absent in the phenylalanine mutant, does not interact directly with imatinib but participates in the closing of the P-loop, which is necessary for trapping the drug inside the kinase domain [25]. Therefore, bonds that are not directly involved in the interaction between the protein and the ligand could have quite a pronounced effect on binding affinity through their role in the stabilization of a particular protein conformation.

Another example comes from the streptavidin-biotin complex. Introducing a disulfide bridge into streptavidin loop 3–4, which participates in the entrapment of biotin, has produced molecules at which the binding affinity is redox switchable [66]. The reduction in this disulfide bond increases *k_off_* by 19,000-fold [66]. However, the crystal structures have shown that the introduced cysteine residues, which form the disulfide bridge, do not directly interact with biotin but instead stabilize the closed conformation of loop 3–4 [66]. Hence, the conformation of loop 3–4 significantly impacts the binding affinity of biotin.

If the binding affinity was solely determined by the direct interactions between the ligand and the protein, it would be anticipated that during competitive inhibition, the inhibitor should establish a greater number or stronger bonds in the enzyme’s catalytic center compared to the substrate of the reaction. However, surprisingly, this is not always the case. For instance, the crystal structures of NMT complexes show that the substrate (peptide) engages in a greater number of and more intense interactions within the catalytic center compared to the NMT inhibitors IMP-1088 and DDD85646 [14]. Similar observations could be made for the binding of ATP and ATP-competitive inhibitors in kinases (unpublished results). These observations can be explained through the ligand-trapping model. While the enzyme successfully releases the reaction products by transitioning to an open conformation, inhibitors become entrapped in the closed enzyme conformation and cannot dissociate, thereby impeding the binding of new substrate molecules. Thus, the competition between the substrate and the inhibitor is not determined by which one forms the strongest bonds with the enzyme but rather by which molecule is more efficiently entrapped.

### 5.4. The Bonds Participating in the Entrapment Have a Dramatic Effect on the Binding Affinity 

In the lock and key model, the formation of a single bond should contribute according to its strength to the overall binding activity (i.e., according to the free binding energy released during its formation). For a single methyl group, the contribution to affinity is expected to be in the range of 3–3.5 fold, and for a hydrogen bond, not to exceed 10 fold, depending on the protein-ligand complex [65]. However, the bonds that participate in the trapping of the ligand have an unexpectedly high contribution to binding affinity. For example, the addition of a single methyl group to an inhibitor of p38α kinase increases the binding affinity by 208-fold, and molecular dynamic simulations have shown that the role of the methyl group is to stabilize the closed protein conformation [25,64,65]. Molecular dynamic simulations have shown that in the absence of the methyl group, the P-loop, which is involved in the trapping of the drug, transitions to an open conformation, releasing the trap mechanism [25]. Hence, the role of the methyl group is to stabilize the inhibited closed protein conformation. Similar results were obtained for the methyl group of imatinib in its complex with Abl kinase [25]. In the literature, numerous examples can be found where the addition of a single methyl group dramatically increases binding affinity and potency, often by hundreds or even thousands of folds [65,69]. Due to the highly unanticipated effect on the methyl group, this phenomenon has become known as a “magic” methyl effect and has been observed in diverse proteins besides the kinase family, including other enzymes and G-protein-coupled receptors [65,69,85]. The role of the methyl group in these examples could be to enhance the entrapment of the inhibitors. However, this phenomenon is not unique to the methyl group, and a similar disproportionately high contribution to affinity has been seen for other bonds, including salt bridges, hydrogen bonds, halogen bonds, and stacking interactions [86]. For instance, in NMT, the formation of a salt bridge between the inhibitors and the carboxyl terminus of the enzyme increases the potency by over 1300-folds, and in the epidermal growth factor receptor (EGFR), a salt bridge with the critical Asp831 boosts the activity of an inhibitor by over 800-fold [82,86,87]. Moreover, a thrombin inhibitor shows a remarkable increase in activity of over 500-fold through the addition of an ammonium group, which participates in a single hydrogen bond in the catalytic site of the protease, and a stacking interaction increases the potency of an inhibitor of the soluble epoxide hydrolase (sEH) inhibitors by 100-fold [86].

**Figure 8:**
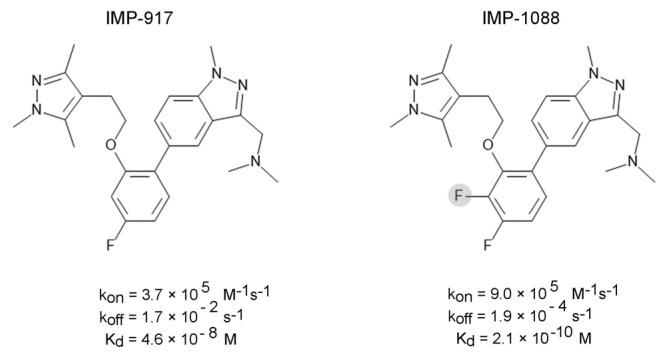
The structures of IMP-917 and IMP-1088. A gray circle indicates the extra fluorine atom in IMP-1088. The fluorine atom reduces *k_off_* by approximately 90-fold and increases mildly *k_on_* by 2.43-fold, leading to a net increase in binding affinity of 219-fold.

In the *N*-myristoyltransferases, the addition of a single fluorine atom boosts the inhibitor’s affinity by 219-fold [47] (Figure 8).

Kinetics analysis has revealed that this is accompanied by a remarkable reduction in the dissociation rate constant, *k_off_*, by approximately 90-fold, while the association constant, *k_on_*, is increased only 2.43-fold (Figure 8) [47]. Therefore, while the contribution of the fluorine atom to the binding step aligns with the estimated change in free binding energy, its role in enhancing the entrapment of the inhibitor leads to a pronounced effect on binding affinity. 

The effect of these changes on affinity is strictly position dependent—similar substitutions elsewhere in the small molecules do not result in such dramatic differences [65]. Therefore, only specific bonds contribute to the trapping effect. As a result, in a given protein-ligand complex, some bonds may contribute only slightly to binding affinity, while others can significantly enhance it, even if they are of the same type or strength. Despite numerous examples of such bonds in the literature, their mechanism has not been explained or linked to the entrapment of the ligand molecule until recently. Our studies of NMT and protein kinases suggest that the presence of such bonds is an indicator of the existence of a trapping mechanism within the studied protein-ligand complex [14,25]. This highlights how minor alterations to the inhibitor, such as adding a single methyl group, a halogen atom, or a hydrogen donor or acceptor, can profoundly affect the binding affinity. The challenge in drug design lies in predicting where these groups should be placed to enhance the binding affinity of the inhibitors.

### 5.5. Effect of Water Exclusion on Binding Affinity

The entrapment of the ligand inside the protein leads to its insulation from the outside aqueous solution. This can be clearly seen in the crystal structures of the complexes of hexokinase, NMT, Abl, RAS, and streptavidin (Figure 3, Figure 4, Figure 5, Figure 6 and Figure 7), and it is likely a general characteristic of the mechanism. In the crystallographic structures of imatinib complexes, the drug is less exposed to the solvent when in a complex with Abl, compared to its complex with Src, consistent with its level of entrapment and binding affinity (Figure 6). This suggests the hypothesis that the exclusion of water from the binding site may play a critical role in mediating the effects of the entrapment on the dissociation rate and overall binding affinity.

Indeed, water molecules can actively participate in hydrogen bonds with polar chemical groups in the protein binding site and the ligand, preventing the interaction between them [88]. It is well known that hydrogen bonds or salt bridges exposed to solvent, such as those on the surface of proteins, have little effect on protein-ligand binding because they can interact with the nearby water molecules [89,90,91]. In contrast, those buried in the hydrophobic environment of the protein structure contribute significantly to binding [89,90,91]. While the general effects of solvation and desolvation are currently understood [92], the inhibitor trapping model suggests that these effects could depend on the protein conformation and specifically on the establishment of a closed protein state, wherein the water molecules are excluded from the binding site. Conversely, the opening of the protein structure may lead to an influx of water into the binding site, disrupting the bonds between the ligand and the protein and dissociating the ligand. In support of the above statements, water displacement from the binding site due to the formation of a protein-ligand complex has been experimentally observed in high-resolution neutron structures of complexes between trypsin and *N*-amidinopiperidine and benzamidine inhibitors, for example [88].

Since the exclusion of water from the binding site ultimately depends on the protein’s structure and topology, bonds that stabilize specific protein conformations can significantly contribute to binding affinity, even if they are not directly formed between the ligand and the protein (see Section 5.3). This process may also explain why certain bonds have a dramatic impact on the ligand’s binding affinity (see Section 5.4). For instance, by stabilizing the closed protein conformation, such bonds could limit the access of water molecules to the binding site, substantially reducing the ligand’s dissociation. 

Water exclusion from the binding site could be fundamentally required for the formation of stable protein-ligand complexes [93], so in general, the trapping mechanism may play an indispensable role in attaining high-affinity binding. These aspects are overlooked in the lock and key, induced fit, and conformational selection theories, in which the binding site is assumed to be always open and accessible to the ligand and solvent (see Figure 1). Clearly, these models do not account for the significant role of hydrogen bonds and salt bridges in protein-ligand interactions, as their contribution to an aqueous solution should be minimal.

### 5.6. Conformational Changes and Protein Dynamics Are Critical for Binding Affinity

The trapping requires the formation of a closed protein conformation, wherein the small molecule is captured inside. The existence of this closed, inhibited state is contingent on low protein dynamics, as increased dynamics could lead to the opening of the structure and dissociation of the ligand. Experimental evidence for this notion has been reported. For instance, relaxation dispersion techniques have revealed that the dissociation rate and overall affinity constant of the dihydrofolate reductase (DHFR) complexes with a number of antifolate inhibitors are predominantly influenced by the enzyme conformational movements [74]. Similarly, single-molecule kinetics analysis has revealed that the dissociation of maltose from its complex with maltose-binding protein is primarily driven by the dynamic conformational changes of the protein [94].

In NMT, molecular dynamics simulations have revealed that the potent nanomolar inhibitors IMP-1088 and DDD85646 reduce the conformational dynamics the most, compared to 24 other ligands with modest micromolar activities, and that the conformational changes in the complex could be correlated with the potency of the compounds [14]. Therefore, potent inhibition arises as a result of imposing a conformational “freeze” on the protein structure, effectively trapping it in a specific conformation. This phenomenon is evident in the examination of various crystal structures, where the protein consistently adopts a different singular conformation depending on the structure of the inhibitor. For example, Abl crystallizes in the inactive DFG-out conformation in complex with imatinib and in the active DFG-in conformation with dasatinib [84,95]. The epidermal growth factor receptor (EGFR) adopts the inactive αC-helix-out conformation in complex with lapatinib and the active αC-helix-in conformation in the presence of erlotinib [79,96]. These, and many other similar observations in kinases and other proteins, have led to the formulation of the conformation selection theory, according to which the inhibitor selects and binds a particular protein conformation. However, considering that the proteins can transition between different conformations, the inhibitor must not only recognize a specific conformation but also to effectively trap the protein in that particular conformational state. What the conformational selection theory fails to recognize is that during the interaction, the small molecule itself becomes entrapped within the structural confines of the protein structure. Consequently, the trapping of the inhibitor may impose the observed block in the protein conformational dynamics. Hence, the inhibitor-trapping mechanism exhibits dual effects: as the inhibitor becomes entrapped within the protein structure, the protein structure itself becomes “locked” into a specific conformational state.

### 5.7. Inhibitor Trapping and Fragment-Based Drug Design

The concept of inhibitor trapping was developed based on the observed synergism between fragments during the development of the NMT inhibitors [14]. For example, IMP-1088 was designed using a fragment-based approach by joining two fragments—IMP-72—that bind to the C-terminus of the enzyme, which is deeply buried in the enzyme active site, and IMP-358, which binds near the Ab-loop of NMT [47]. Interestingly, when these two fragments are combined in an enzymatic assay, their inhibitor activity is increased over 300-fold [47]. Such behavior was difficult to explain through the lock and key, induced fit, or conformational selection models [14]. For example, in the lock and key model, the effect of combining two fragments on inhibition is expected to be additive (not highly synergistic), and the crystal structures of NMT complexes with the fragments do not reveal conformation changes in the NMT to be consistent with induced fit or conformational selection [14]. However, molecular dynamics simulations have shown that one of the fragments (IMP-358) prevents the opening of the Ab-loop in NMT, effectively trapping both fragments inside its catalytic center [14]. As a result, the dissociation rate of IMP-72 is reduced by the presence of IMP-358, leading to enhanced inhibition [14]. 

Overall, the success of fragment-based drug design may depend on obtaining synergism between the joined fragments. A typical example is when two fragments with modest micromolar activity are joined together to produce a nanomolar inhibitor. Such is the case with an inhibitor of the matrix metalloproteinase stromelysin (MMP-3) [97] and many other cases where a fragment-based approach has been successful [97,98,99,100]. Hence, the concept of inhibitor trapping may prove critical for understanding fragment-based drug design.

### 5.8. Inhibitor Trapping and Allostery

Most examples of inhibitor trapping in this article are cases of competitive inhibition, where the inhibitor binds to the same binding site as the substrate of the reaction. However, it is possible that trapping plays a key role in other types of enzyme inhibition. In noncompetitive inhibition, the inhibitor binds to a site distinct from the enzyme catalytic site (allosteric site) and exerts its effect through a conformational change in the enzyme structure [101]. The effect of these allosteric inhibitors could depend on blocking the conformational transitions in the enzyme—for example, its opening and closing or the formation of the catalytically competent state. Theoretically, an allosteric inhibitor could inhibit the enzyme by stabilizing it in the closed conformation with the substrate trapped inside or by preventing the binding of the substrate in the active center. Stabilizing the open enzyme conformation could also be inhibitory because it will prevent the formation of the closed catalytically competent state. Hence, trapping the enzyme in a specific conformation may serve as a key mechanism for allosteric regulation, as well. In fact, in the preceding subsection, it was described how, in NMT, one fragment could augment the activity of another through entrapment, demonstrating how trapping can manifest allosteric effects [14].

## 6. Predicting Binding Affinity

### 6.1. Models of Molecular Recognition in Computational Drug Design

The existing computational tools for binding affinity prediction are based on the lock and key, induced fit, and conformational selection models of protein-ligand recognition but do not account for the inhibitor entrapment mechanism because the latter was only recently introduced [14,25]. For instance, docking, in its simplest versions, is based on the lock and key model. Typically, during docking, the protein structure is regarded as rigid, and the aim is to find the best fit between the ligand and the protein, akin to the lock and key analogy [10,17]. Some docking programs, such as GOLD, allow modeling of some aspect of induced fit “hand in glove” binding by permitting side chain flexibility of the amino acid residues in the binding site [102]. Other docking programs model conformational movement in the protein backbone during binding, a technique that has gained traction in protein-protein docking [103,104]. However, these programs are largely unable to model large conformational changes, such as hinge domain movement, that may occur during induced-fit binding. Such large conformational changes upon ligand binding can be successfully modeled by using molecular dynamics simulations [105]. Ensemble docking, which allows different protein conformations to be used simultaneously for docking, attempts to model the conformational selection theory [106]. However, because neither of these approaches provides information about ligand dissociation, they alone cannot reliably predict binding affinity. 

Given that the model of inhibitor entrapment was only recently proposed, computational methods specifically designed for this process have yet to emerge. Among current computational methods, only molecular dynamics simulations can provide insights into inhibitor entrapment. In studies involving NMT and kinases, MD simulations have demonstrated the ability to predict conformational transitions from closed to open enzyme conformations and subsequently assess trapping and binding affinity [14,25]. Notably, in NMT, a correlation was identified between the stability of the closed enzyme conformation and inhibitor potency [14]. Observing the conformational changes that lead to the opening or closing of a protein requires relatively long molecular dynamics simulations, lasting for at least 1 µs [14,25]. This makes the process computationally and time intensive. Therefore, there is a need for novel computational techniques capable of analyzing entrapment in protein-ligand complexes. 

### 6.2. A Unified Theory of Protein-Ligand Interaction

Since new theories build on the foundations of older ones, the latest theories of molecular recognition are inclusive of the earlier versions [30]. For example, the lock and key model can be considered as a special case of induced fit, where there is no conformational change during binding, or a case of conformational selection, where there is only one pre-existing protein conformation. Similarly, lock and key, induced fit, and conformational selection can be considered as special cases of ligand trapping, where the degree of entrapment is negligible, as this mechanism has not been under consideration in these previous theories.

**Figure 9:**
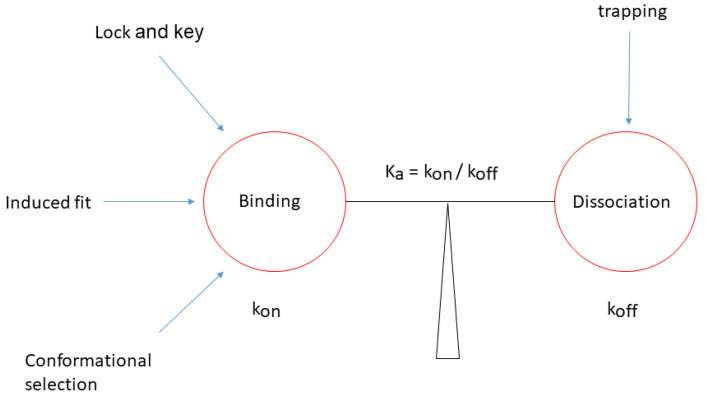
A unified theory of protein-ligand interaction. The scheme depicts the relations between the models of protein-ligand recognition, binding affinity *K_a_*, and the rate constants *k_on_* and *k_off_*. The lock and key, induced fit, and conformational selection describe the ligand binding to the protein, while the trapping model describes its dissociation. Binding affinity prediction depends on estimating both the binding and dissociation rates.

In this context, the theory of inhibitor trapping is the most comprehensive and could unify previous molecular recognition models into a general theory of protein-ligand interaction. (Figure 9). In this theory, the lock-and-key, induced fit, and conformational selection models describe ligand binding, while inhibitor trapping addresses ligand dissociation (Figure 9).

Binding affinity results from the equilibrium between the binding and dissociation rates (see Section 2); hence, evaluating both is essential for determining binding affinity. As such, the theories describing ligand binding (lock-and-key, induced fit, conformational selection) and those elucidating dissociation (inhibitor trapping) are both necessary for accurate binding affinity prediction. In this regard, the models that describe the binding (association) of the ligand are incomplete and insufficient for predicting binding affinity. This is because the dissociation rate constant *k_off_*, has a profound effect on the overall binding affinity (see Table 1). For example, the entrapment of biotin increases its binding affinity over 3.7 million-fold, of nilotinib over 12,000-fold, of imatinib over 1700-fold, and of the NMT inhibitor—IMP-1088 over 5200-fold (Table 1). Such substantial differences in computed and experimental values of the affinity constant are to be expected when the trapping mechanism is not included in the calculations. This is in agreement with our previous studies, where we found that compounds identified by virtual screening were thousands of folds less active against NMT than predicted by computational approaches [13,14]. 

### 6.3. Predicting Binding Affinity 

The contribution to free binding energy occurs not only during binding but also during dissociation of the ligand. Therefore, the successful prediction of binding affinity depends on using methodologies that provide reliable estimations for the binding (association) of the ligand and combining them with approaches that give insights into its dissociation. This differs from the approach in the current docking programs, which tries to approximate binding affinity based only on the association between the protein and the ligand. For example, the docking programs provide affinity scoring functions based on changes in free binding energy [8]. However, since they evaluate binding without accounting for dissociation, these scoring functions may only pertain to the formation of the complex rather than the overall binding affinity. Therefore, these observations suggest that the computational tools aiming to estimate binding affinity need to provide two independent scoring functions—one for ligand binding and another for its dissociation. 

In this aspect, the difficulty of docking in predicting binding affinity is not as much related to the docking program used as to the fact that it fundamentally provides only half of the information that is needed to compute the binding affinity. How accurate the docking is in relation to the experimental values of binding affinity could mostly depend on the protein-ligand complex and whether the ligand is strongly or weakly entrapped. For example, in complexes where entrapment is not very prominent or negligible, the docking scores are expected to be closer to the actual values. In contrast, when comparing ligands that are strongly entrapped with ones that are not, substantial differences between calculated and experimental values are expected [13,14].

Then, how can the binding affinity be estimated? One difficulty arises due to the fact that the binding of the ligand may change the conformation of the protein according to the induced fit or conformational selection theory, and subsequently, this can affect its dissociation rate due to increased or decreased entrapment. In this aspect, the docking programs need to get better at predicting how the binding of a particular ligand affects the conformation of the protein. Currently, the closest to this is the ensemble docking, where different protein conformations can be tested for binding simultaneously. Most docking programs have the capacity for ensemble docking, but it is not clear whether they can test the entire conformational space. Potentially, this can be resolved by combining docking with MD simulations. For example, by using molecular dynamics simulations, it has become possible to model the induced fit binding of imatinib to Abl kinase to obtain the exact closed inhibited enzyme conformation observed in the crystal structure of their complexes [105]. Alternatively, this can be performed by coupling docking programs with artificial intelligence tools trained on databases of structures of protein-ligand complexes, with the idea that they can predict the most likely conformation of the protein and the ligand in a given protein-ligand complex. In this aspect, the success of Alphafold in predicting the 3D structures of the proteins is promising [107]; however, it remains to be seen whether it can predict the changes in the protein conformation resulting from the binding of a ligand. 

Therefore, in the first step, the conformation of the protein and the docked ligand needs to be determined to obtain information about the binding rate constant. Based on this structure, the dissociation rate may be estimated in the next step. From the current methods, only MD simulations can provide insights into the opening or closing of the protein structure and the ligand dissociation rate (see Section 6.1). Computing the potential of mean force (PMF) using the umbrella sampling method from MD simulations may provide valuable insights into the ligands’ dissociation rate and their binding affinity [108]. This method allows performing pulling simulations of the ligand from the protein with minimal conformational restrictions on the protein structure, thus enabling protein conformational changes during ligand dissociation simulations. The calculation of PMF has produced results that are in agreement with experimentally obtained values for binding affinity, for example, for the interaction of piperine with heat shock protein 70 (HSP70) and the cytokine IL-1β [109,110]. In general, the PMF profiles reveal the energetic barrier for unbinding the ligand from the binding site [109,110], which could make this method particularly useful for estimating ligand entrapment. For instance, the ligands that are highly entrapped are expected to have a high energetic barrier to unbinding, compared with the ligands that are weakly entrapped. In this aspect, it will be of interest to perform PMF analysis of protein-ligand complexes that display a high level of entrapment, such as in the examples of tight inhibitors described in this article. 

Since MD simulations are time-intensive, alternative strategies may need to be developed to assess the level of entrapment more efficiently. For example, these new approaches could enable the evaluation of a large number of compound structures during virtual screening or aid in designing structures predicted to achieve high levels of entrapment. Parameters for such analysis could include the degree of openness or closeness of the molecule, the exclusion of water from the binding site, and the dynamic stability of the protein conformation. Artificial intelligence approaches could be used to uncover connections between these or other yet unknown parameters and the binding affinity using the crystal structures of protein-ligand complexes deposited in protein databanks or based on molecular dynamics simulations. As such, the key to successful binding affinity predictions could be the development of approaches to estimate the level of entrapment and its effect on the dissociation rates of the ligands.

## 7. Conclusions

Currently, the accurate estimation of binding affinity remains an elusive goal, with predicted values often diverging by orders of magnitude from experimental results. This represents a significant setback for the development of novel therapeutics, which still depends mostly on experimental efforts, which are time- and resource-intensive. A comprehensive understanding of the mechanism that determines binding affinity can hold the key to the successful design of drugs with high affinity and activity. In this context, the numerous examples described in the article unveil a novel, groundbreaking paradigm for protein-ligand recognition that could redefine our approach to drug design and may have a substantial impact on medicinal chemistry and clinical practice in the future.

## Figures and Tables

**Table 1:** Dissociation and rate constants in some protein-ligand complexes.

	Ligand	Protein	*K_d_*, M	*k_on_*, M^−1^s^−1,^	*k_off_*, s^−1^	Res Time *, s	Ref
1	biotin	streptavidin	5.0 × 10^−15^	5.40 × 10^7^	2.70 × 10^−7^	3,703,704	[66]
2	IMP-1088	NMT	2.1 × 10^−10^	9.00 × 10^5^	1.90 × 10^−4^	5263	[47]
3	imatinib	Abl	3.83 × 10^−9^	1.53 × 10^5^	5.86 × 10^−4^	1706	[70]
4	nilotinib	Abl	2.88 × 10^−9^	2.86 × 10^4^	8.24 × 10^−5^	12,136	[70]
5	dasatinib	Abl	5.0 × 10^−10^	2.27 × 10^6^	1.13 × 10^−3^	885	[70]
6	dasatinib	EGFR	1.28 × 10^−8^	7.81 × 10^5^	1.00 × 10^−2^	100	[71]
7	lapatinib	EGFR	1.22 × 10^−8^	9.79 × 10^4^	1.20 × 10^−3^	833	[71]
8	staurosporine	EGFR	6.00 × 10^−8^	4.93 × 10^5^	3.00 × 10^−2^	33	[71]

* Residence time = 1/*k_off._*
